# The use of graphic organizers in science education of deaf and hard-of-hearing students

**DOI:** 10.3389/fpsyg.2026.1844334

**Published:** 2026-05-22

**Authors:** Necla Işıkdoğan Uğurlu

**Affiliations:** Department of Special Education, Ereğli Faculty of Education, Zonguldak Bülent Ecevit University, Zonguldak, Türkiye

**Keywords:** deaf and hard of hearing students, graphic organizers, inclusive, science education, scientific process skills

## Abstract

**Introduction:**

Deaf and hard-of-hearing (DHH) students experience persistent difficulties in science education due to language and communication barriers, emphasizing the need for effective visual and multimodal instructional approaches. This study investigated the effects of graphic organizer-supported instruction on the scientific process skills (SPS) of DHH students.

**Methods:**

A mixed-methods design was employed with 65 DHH students from primary, middle, and high schools and 6 teachers. Graphic organizer-supported activities were integrated into science instruction. Quantitative data were analyzed using repeated-measures ANOVA, while qualitative data were obtained through semi-structured interviews and analyzed using content analysis.

**Results:**

Findings revealed a statistically significant improvement in SPS across measurement points. Students’ positive attitudes toward science significantly influenced performance, whereas grade level showed no significant effect. Observation skills demonstrated the greatest improvement among SPS dimensions. Qualitative findings supported the quantitative results and highlighted the importance of multisensory instruction, repetition, structured visual supports, and vocabulary development.

**Discussion:**

The findings suggest that graphic organizers are effective in enhancing SPS among DHH students and support the use of visual and multimodal strategies in inclusive science education. Future studies may employ experimental and longitudinal designs to examine long-term effects.

## 1 Introduction

Individuals benefit from non-formal and formal education from early childhood to adapt to the rapidly developing and changing world. The quality education services have fundamental aims such as delivering information to children, adapting acquired knowledge to different settings, and ensuring the functional use of information. The science course constitutes a fundamental part of the education services provided to students. İflazoğlu and Saban (2014) define the fundamental aims of the science course as developing knowledge through collaboration based on students’ skills and interests and enabling the use of these skills in their daily lives. In our country, the Ministry of National Education [MEB] (2018) states that the main purpose of science course is to enable students to become science literate in accordance with their individual differences. Definitions regarding science clearly consist of features such as enabling students to use scientific process skills to solve problems in their daily lives, developing positive attitudes toward science, and acquiring knowledge and skills. Thus, students will be able to better comprehend the world and their immediate environment and adapt to daily life.

MEB (2018), aiming to develop these skills for all students, emphasizes the importance of considering students’ individual differences, interests, needs, competencies, and disabilities. It is also seen that importance is given to the arrangement of learning environments by considering individual differences of students in the implementation of science courses (Kaplan, 2011; Güzel-Özmen et al., 2002). These arrangements and adaptations are of vital importance for DHH students, as they are for all students.

In addition to academic skills and literacy skills, branch courses are also included in the curriculum. In developed countries, such as America, Individualized Education Programs (IEPs) are prepared for children with hearing loss in accordance with their characteristics (Çiftçi, 2009). In our country, the curriculum is similar for all students and a separate program specially designed for DHH students does not exist. Therefore, it is necessary to adapt the curriculum that is common for all students and to arrange course materials and teaching methods. With the legal regulations made in the field of special education and rehabilitation after 2008, the implementation of IEPs for children with hearing loss began to be addressed in our country (Çiftçi, 2009). Considering the importance of science courses in preparing students for life, the adaptation of science courses for DHH students and providing materials are of vital importance (Hall et al., 2019; Knoors and Marschark, 2020). Science courses facilitate DHH students’ daily lives, support their language and conceptual development, ensure the acquisition of a scientific identity and self-confidence, positively affect their attitudes toward science, and support visual learning. Marchut and Gormally (2019), in their study conducted with children with hearing loss, examined the effects of inquiry-based science instruction. The results of their study demonstrated that students with hearing loss developed positive attitudes toward science through this approach (Knoors and Marschark, 2020; Hrastinski and Wilbur, 2016). These considerations underscore the importance of adapting science instruction for DHH students and provide a foundation for exploring the use of graphic organizers as an effective instructional strategy.

For deaf and hard-of-hearing (DHH) students, access to quality science education presents unique challenges related to language, communication, and instructional design. Research has consistently shown that language accessibility is a central factor influencing cognitive development and academic achievement in DHH learners (Hall et al., 2019; Marschark et al., 2015). In addition, family involvement and early educational experiences significantly shape children’s adaptation to school environments. Recent evidence highlights that parental perspectives, communication practices, and school readiness play a critical role in the school-starting process and overall educational trajectories of DHH children (Işıkdoğan Uğurlu et al., 2026). These factors underscore the importance of designing accessible and language-sensitive instructional approaches that can effectively support the academic and cognitive development of DHH learners.

It is observed that there is limited research in the literatüre regarding the DHH students’ acquisition of scientific process skills in science courses (Ediyanto, 2018; García-Terceño et al., 2023; Raven and Whitman, 2019). In the educational services provided in schools, factors such as limited resources, negative attitudes of teachers, language of instruction (sign language/verbal language), and teaching methods can negatively affect DHH children’s motivation and attitudes. Therefore, this situation can lead to negative effects on academic success and social development (McGinnis and Kahn, 2014). As in the rest of the world, in our country, it is necessary to prioritize bilingual (sign and verbal language) communication, enrichment of education services, and inclusiveness to establish fully accessible, equitable, and inclusive environments for DHH children (State Confederation of Deaf People [CNSE], 2021; Cannon et al., 2022). In this context, structured and visually supported instructional approaches, such as graphic organizers, may offer a promising way to address these challenges.

Given the difficulties encountered by DHH students due to hearing loss, visual aids are of great importance for benefiting from educational services. As in all academic courses, visual resources are frequently used in science courses for introducing concepts, explaining instructions, and developing comprehension skills. These can be presented through various ways, such as pictures, videos with written and sign language, graphic organizers, visuals on the boards (Lomber, 2017; Dexter and Hughes, 2011). In particular, graphic organizers enable simultaneous monitoring of various skills in science courses, such as inquiry, comparing prior knowledge with new information, and advanced thinking skills (Mastropieri et al., 2006). Scruggs et al. (2017) suggest that graphic organizers provide many benefits for students and highlight that they have positive effects on planning education services appropriate for students’ characteristics, acquisition of subject content, and retention of knowledge.

Recent studies have demonstrated that visual scaffolding techniques, including graphic organizers, significantly improve learning outcomes for students with diverse learning needs, including those with disabilities (García-Terceño et al., 2023). In science education contexts, graphic organizers have been associated with improved conceptual understanding, retention, and engagement (Novak and Cañas, 2008). Furthermore, multimodal approaches that integrate visual, linguistic, and experiential components have been shown to enhance participation and learning outcomes among DHH students (García-Terceño et al., 2023).

Despite these advances, the literature reveals a persistent gap regarding the development of scientific process skills among DHH students. Recent studies have increasingly emphasized the role of multimodal and visually enriched instructional approaches in supporting the academic development of DHH learners. For instance, recent research highlights that integrating visual scaffolds with inquiry-based practices improves both conceptual understanding and engagement in science learning environments (García-Terceño et al., 2023; Cannon et al., 2022). Additionally, emerging evidence suggests that technology-supported visual tools and structured representations further enhance accessibility and participation for DHH students in inclusive classrooms (Knoors and Marschark, 2020). These findings reinforce the importance of designing instruction that aligns with the visual learning strengths of DHH learners. Much of the existing research focuses on language development or general academic achievement, while relatively few studies specifically examine SPS development in science education contexts (Freeman et al., 2014; Graham, 2012; Raven and Whitman, 2019; Marschark et al., 2015). Additionally, teachers o’en report challenges in implementing inquiry-based and student-centered approaches due to limited training, resources, and adapted instructional materials (Hrastinski and Wilbur, 2016; Santana and Sofiato, 2018). Given the importance of SPS in fostering scientific literacy and the identified gaps in the literature, there is a need for research that explores effective instructional strategies tailored to DHH learners. Graphic organizers offer a promising approach by providing structured visual representations that align with the visual learning strengths of DHH students while supporting cognitive processing and conceptual understanding.

This study is grounded in Dual Coding Theory (Clark and Paivio, 1991; Paivio, 1986), which posits that information is processed through two interconnected cognitive systems: a verbal system and a non-verbal (visual) system. Learning is enhanced when information is presented through both channels, as this dual representation strengthens memory and facilitates deeper understanding.

In science education, graphic organizers function as visual tools that structure relationships between concepts, enabling learners to organize, compare, and interpret information more effectively. This is particularly important for deaf and hard-of-hearing (DHH) students, who rely more heavily on visual input due to limited access to auditory information (Marschark and Wauters, 2008). From a dual coding perspective, integrating visual representations with verbal or signed explanations reduces cognitive processing demands and supports meaningful learning. Graphic organizers allow DHH learners to simultaneously access visual and linguistic information, thereby enhancing conceptual understanding and scientific reasoning. Accordingly, the present study assumes that instruction supported by graphic organizers facilitates the development of scientific process skills by strengthening the interaction between visual and verbal cognitive systems. This theoretical grounding provides a framework for explaining how and why graphic organizers may improve learning outcomes in DHH students.

Therefore, this study aims to examine the effectiveness of instruction supported by graphic organizers in enhancing the scientific process skills of DHH students in science education. Specifically, the study addresses the following research questions:

RQ1: What is the impact of graphic organizers on the overall development of scientific process skills of students (DHH) in a science course across three measurement points?

RQ2: What is the impact of graphic organizers on the development of scientific process skills dimensions (observation, classification, communication, measurement, prediction, and interpretation) of students (DHH) in a science course across three measurement points?

RQ3: Does the development of scientific process skills in students (DHH) who are exposed to graphic organizers differ significantly according to their school level (elementary, middle, high school)?

RQ4: What are teachers’ perceptions regarding the activities implemented after the intervention?

## 2 Method

### 2.1 Participants

The study sample consisted of 65 students (DHH) and 6 classroom teachers. Demographic information about the DHH sample is given in Table 1.

**Table 1:** Demographic and hearing loss characteristics of the DHH sample.

Characteristic	Category	*f*	%
School level	Elementary school	15	23.1
	Middle school	30	46.2
	High school	20	30.8
Gender	Female	28	43.1
	Male	37	56.9
Grade level	3rd grade	9	13.8
	4th grade	6	9.2
	6th grade	15	23.1
	7th grade	15	23.1
	9th grade	10	15.4
	10th grade	10	15.4
Age at diagnosis	Newborn	52	80.0
	3 Years	7	10.8
	4 Years	2	3.1
	Other (2, 5, 6 month, 7 years)	4	6.2
Type of hearing loss	Severe hearing loss	43	66.2
	Profound hearing loss	22	33.8
Assistive device	Cochlear implant	46	70.8
	Hearing aid	2	3.1
	Cochlear implant + Hearing aid	17	26.2
Device usage	Regular	60	92.3
	Irregular	5	7.7
In-class language preference	Sign language	29	44.6
	Oral language	6	9.2
	Total communication	30	46.2
Family member with disability	Yes	35	53.8
	No	30	46.2

Based on the principle of voluntary participation, two primary school (T1, T2), two secondary school (T3, T4), and two high school teachers (T5, T6), six teachers in total (T1, T2. T6) were interviewed in the study. The teachers were those who taught DHH students in the study group and acted as observers/assistants in the activities conducted. All teachers participating in the study had a minimum of five (5) years’ experience.

The participants were recruited from different school levels, including elementary school (*n* = 15, 23.1%), middle school (*n* = 30, 46.2%), and high school (*n* = 20, 30.8%). The sample comprised 37 male (56.9%) and 28 female (43.1%) students. The participants’ ages ranged from 9 to 16 years old (M = 12.86, SD = 2.34). The grade levels of the participants were distributed as follows: 3rd grade (*n* = 9, 13.8%), 4th grade (*n* = 6, 9.2%), 6th grade (*n* = 15, 23.1%), 7th grade (*n* = 15, 23.1%), 9th grade (*n* = 10, 15.4%), and 10th grade (*n* = 10, 15.4%).

Regarding the characteristics of hearing loss, the vast majority of participants (*n* = 52, 80.0%) were diagnosed in the newborn period. The average age at which students started receiving special education services was 5.15 years (SD = 1.23), with a mode of 5 and 6 years (each *n* = 18, 27.7%). The most common type of hearing loss was severe hearing loss (*n* = 43, 66.2%), followed by profound hearing loss (*n* = 22, 33.8%).

In terms of auditory technology use, most participants used a cochlear implant (n = 46, 70.8%). Bilateral device use was common (*n* = 40, 61.5%), and the vast majority used their devices regularly (*n* = 60, 92.3%). The average age of first device fitting was 2.95 years (SD = 1.23). The duration of receiving special education support ranged from 3 to 15 years (Mode = 4 years, *n* = 15, 23.1%). In the present study, detailed audiological measures of functional hearing capacity were not the primary focus. Instead, participants’ auditory profiles were described based on assistive device type, cochlear implant use, and bilateral hearing loss status. It should be noted that students using cochlear implants typically have severe (71–90 dB) to profound (≥91 dB) hearing loss. Therefore, the sample largely represents individuals within these categories, and the degree of hearing loss (severe and profound) was considered as an alternative indicator of hearing ability.

Communication practices within the sample were diverse. During lessons, 30 students (46.2%) used total communication, 29 (44.6%) used sign language, and 6 (9.2%) used spoken language. Family communication outside of school primarily involved sign language (*n* = 37, 56.9%) or total communication (*n* = 27, 41.5%), with only one family relying solely on spoken language. A notable majority of the participants (*n* = 35, 53.8%) had another family member with a communication disability.

The study employed a purposive sampling strategy, specifically criterion sampling, to select participants who met predefined inclusion criteria (Patton, 2015). The criteria included being diagnosed with hearing loss, actively attending a formal educational institution, and being able to participate in instructional activities supported by visual materials. Schools were selected based on accessibility and willingness to participate in the study. This approach ensured that participants were information-rich cases suitable for examining the effectiveness of graphic organizer-supported instruction in DHH students.

### 2.2 Procedures

*Data collection tools*: In the study, a general information form consisting of DHH students’ demographic information, Attitude Toward Science Scale, Scientific Process Skill (SPS) observation form, and a semi-structured interview form to obtain teachers’ opinions about the implementation were utilized.

*General information form:* It is the form that consists of DHH students’ general characteristics, grade levels, health information, language and communication characteristics, family history with hearing loss, and information on the hearing aids they used.

*Attitude toward science scale:* It was developed by Özcan and Koca (2020). The scale consists of 36 items on a 5-point Likert type (1 = never/cannot do it - 5 = always does it) and its Cronbach Alpha coefficient is 0.93. It is the observation form developed by the researcher that consists of fundamental scientific process skills. It evaluates the extent to which the skill was observed.

*Scientific process skill (SPS) observation form:* Within the scope of science, fundamental scientific process skills are addressed under the headings of observation, classification, measurement, data recording, establishing number and space relationships, prediction (forecasting), inference, and scientific communication (Arslan & Tertemiz, 2004; National Academies of Sciences, Engineering, and Medicine [NASEM], 2020; Yapıcıoğlu, 2021). In this study, an observation form consisting of fundamental scientific process skills that was developed by the researcher was utilized to evaluate the extent to which these skills were observed (The skills were rated as observed, acceptable, and need improvement).

*Teacher interview form:* The form utilized to obtain teachers’ opinions about the activities conducted consisted of seven (7) questions. The questions prepared with the support of three (3) field experts investigated how DHH students used graphic organizers in the activities, their opinions about SPS skills, and how to enhance their effectiveness in education environments.

### 2.3 Validity and reliability

To ensure the validity and reliability of the data collection tools, several procedures were implemented. First, the Attitude Toward Science Scale demonstrated high internal consistency, with a Cronbach’s alpha coefficient of 0.93 (Özcan and Koca, 2020). For the Scientific Process Skills (SPS) Observation Form developed by the researcher, content validity was established through expert review. Three experts in science education and special education evaluated the items in terms of relevance, clarity, and appropriateness for DHH students, and necessary revisions were made based on their feedback.

To ensure reliability, inter-rater reliability was considered during the observation process. Observations were conducted systematically, and consistency in scoring was maintained through predefined criteria. Additionally, the qualitative data obtained from teacher interviews were analyzed using content analysis, and credibility was enhanced through prolonged engagement and expert validation (Creswell and Creswell, 2022). These procedures contributed to the trustworthiness and rigor of the study findings.

### 2.4 Data collection

The researcher visited the schools where DHH students attended and conducted interviews with the school administration and teachers. She gave information about the purpose and implementation of the study. Consent for voluntary participation was obtained from DHH students’ families and teachers. Before the implementation, interviews were conducted with students and their approval for voluntary participation was obtained. The demographic information of the students was obtained from the school counseling service, teachers, families, and children.

In the implementation stage of the study, the students were asked to complete the *Attitude Toward Science Scale* first. The Attitude Toward Science Scale was originally developed for school-aged children and consists of clear, concrete, and behavior-oriented items (Özcan and Koca, 2020). To ensure developmental appropriateness across the wide age range (9–16 years), the administration process was adapted for younger participants. Items were explained using simplified language and supported with sign language when necessary. In addition, classroom teachers were present during administration to facilitate comprehension without influencing responses. These adaptations are consistent with recommendations for assessing attitudes in younger and DHH populations, where linguistic accessibility plays a critical role in valid measurement (Hall et al., 2019). Therefore, rather than modifying the instrument itself, procedural adaptations were employed to support comprehension across developmental levels. Since the researcher has a sign language certificate, she made explanations in sign language whenever students had difficulty understanding throughout the study. After all students had completed the attitude scale individually, SPS activities were initiated. SPS activities were conducted under three practices. The SPS checklist was completed by the researcher in each activity. To ensure systematic observation across all 65 participants from different grade levels, students were organized into small groups (pairs) within their own classrooms, and each session followed a structured schedule. Observations were conducted during regular class hours, with each session lasting two class periods. The researcher focused on one group at a time while simultaneously monitoring the overall classroom process. Each scientific process skill (SPS) dimension was evaluated using a structured observation checklist, and observations were recorded immediately during or at the end of each activity to ensure accuracy. To maintain consistency across sessions and participants, the same activity procedures, materials, and instructions were applied in all groups. In addition, classroom teachers were present to support classroom management and ensure continuity, but they did not intervene in the scoring process. This structured and repeated observation process enabled the researcher to collect comparable data across different grade levels and time points. Among graphic organizers, the KWL (know, want to know, learned) form and the comparison and contrast form (common and different characteristics) were utilized in the activities. Students were engaged in pairwork. The students’ classroom teachers were also included in the activities to prevent any possible inconveniences. The study was conducted in 4 weeks; 2 days a week for two course hours. The researcher planned and conducted all activities in line with the SPS checklist. After the activities, meetings were conducted with students’ classroom teachers and science teachers. These meetings were planned outside of course hours.

### 2.5 Characteristics of the activities

The activities were planned in accordance with the science curriculum and grade levels. Chairs and desks were arranged to enable pairwork. The materials were organized separately and placed on the desks for each group. The researcher acted as a role model for students about how they would carry out the activities in all stages. She explained how to use graphic organizers. She guided the students whenever they had difficulty in understanding the activity. Then, she asked students to do the activities individually. SPS dimensions were applied in each activity. The researcher guided all activities; she provided explanations when the students had difficulty understanding, gave examples and repeated if necessary. At the end of the activities, the products were displayed in the classroom and reviewed mutually. The number of materials was increased in higher grade levels. Therefore, materials used in grouping and classification activities in higher grades displayed variety in color, function, texture, size, and number. The activities and their features are shown in Table 2.

**Table 2:** Activities and their features.

Activity	SPS dimensions	Materials	Student product/output
Colorful candies	Observation, classification, communication, measurement, prediction, interpretation	Colorful candies, pens, graphic organizers, cardboards	Preparing graphics for numbers and colors, bar graph, exhibition
Whose cookie is bigger?	Observation, classification, communication, measurement, prediction, interpretation	Cookies, cardboards, pens, graphic organizers, papers	Praparing graphics according to the sizes of cookies, exhibition
Which is the heaviest box?	Observation, classification, communication, measurement, prediction, interpretation	Boxes, various materials for weight, graphic organizers, pens	Preparing graphics according to weight, exhibition

### 2.6 Statistical analyses

The study addressed three primary research questions. The first question (RQ1) focused on the impact of graphic organizers on the overall development of scientific process skills (SPS) in students (DHH). To answer this, a one-way repeated-measures analysis of variance (ANOVA) was conducted with Time (pre-intervention/SPS1, during intervention/SPS2, post-intervention/SPS3) as the within-subjects factor. Mauchly’s Test was used to verify the sphericity assumption, and Bonferroni-adjusted pairwise comparisons were applied following any significant main effects to pinpoint differences between specific time points.

The second question (RQ2) sought to determine the impact on specific SPS dimensions, namely observation, classification, communication, measurement, prediction, and interpretation. A series of separate repeated-measures ANOVAs were performed for each skill dimension. For observation skills, which violated the normality assumption, the non-parametric Friedman test was utilized instead, followed by appropriate post-hoc analyses.

The third question (RQ3) examined whether skill development differed according to grade level (elementary, middle, high school). This was analyzed using a mixed-design ANOVA, with Time as the within-subjects factor and School Level as the between-subjects factor, while controlling for student attitude as a covariate. Attitude was included as a covariate to control for its potential influence on students’ performance and to better isolate the effect of the intervention on SPS development. The analysis included checks for sphericity (Mauchly’s Test), homogeneity of covariance (Box’s M Test), and homogeneity of error variances (Levene’s Test) (Glass and Hopkins, 1996; Hair et al., 2019; Howell, 2013). Although Box’s M test indicated a violation of the homogeneity of covariance matrices assumption, the analysis proceeded given that the sample sizes across groups were relatively balanced and the analysis of variance is considered robust to moderate violations of this assumption (Hair et al., 2019).

All analyses were performed using IBM SPSS Statistics 27. The practical significance of findings was interpreted using partial eta-squared (partial *η*^2^) effect sizes, following Cohen (1988) conventions, where values of 0.01, 0.06, and 0.14 represent small, medium, and large effects, respectively.

Following the quantitative findings obtained from these analyses, qualitative data were collected to substantiate the results within the scope of the fourth research question: “RQ4: What are teachers’ perceptions regarding the activities implemented after the intervention?” Subsequently, content analysis was conducted on these qualitative data.

## 3 Results

### 3.1 Impact of graphic organizers on the overall development of scientific process skills of DHH students

The impact of graphic organizers on the development of scientific process skills (SPS) of students with hearing loss (DHH) in a science course was determined using a repeated-measures analysis of variance (ANOVA). A repeated-measures ANOVA (mixed-design) was employed to examine the change in scientific process skills (SPS) measured at three time points and the effect of the attitude factor on this change. The mean and standard deviation values of the SPS scores for the 65 participants are presented in Table 3.

**Table 3:** Descriptive statistics for scientific process skills (SPS) scores.

Time point	Mean	Standard deviation	Skewness	Kurtosis
SPS1	39.06	11.96	0.314	−1.020
SPS2	44.66	12.13	−0.225	−1.166
SPS3	56.23	6.92	−0.953	0.537

Mauchly’s test was conducted to determine whether the sphericity assumption was met. The test result indicated that the sphericity assumption was not violated, *χ*^2^(2) = 0.55, *p* = 0.759; therefore, no correction to the degrees of freedom was necessary. As provided in Table 4, the repeated-measures ANOVA results revealed that the time factor had a statistically significant main effect on SPS scores, *F*(2, 126) = 11.35, *p* < 0.001, partial *η*^2^ = 0.15. This finding indicates that 15% of the variance in SPS scores is explained by the time factor. The time x attitude interaction was found to be marginally significant, *F*(2, 126) = 2.94, *p* = 0.054, partial *η*^2^ = 0.05. This result suggests a trend wherein the developmental profiles of SPS over time may differ across attitude levels, though this interaction did not reach conventional levels of statistical significance.

**Table 4:** Repeated-measures ANOVA results - within-subjects effects.

Source	Sum of squares	df	Mean square	*F*	*p*	Partial *η*^2^
Within subjects						
Time	878.17	2	439.09	11.35	<0.001	0.15
Time × attitude	230.74	2	115.37	2.98	0.054	0.05
Error (time)	4,873.48	126	38.68			
Between subjects						
Intercept	121,190.46	1	121,190.46	47.59	<0.001	0.43
Attitude	383.44	1	383.44	1.50	0.226	0.02
Error	16,138.18	63	256.16			

The between-subjects analysis results showed that the main effect of the attitude factor on SPS scores was not statistically significant, *F*(1, 63) = 1.50, *p* = 0.226, partial *η*^2^ = 0.02. This finding indicates that the overall performance levels of SPS were similar across different attitude levels. Since the main analysis for the time factor was significant, pairwise comparisons with Bonferroni correction were conducted to determine between which specific time points the significant differences lay. The results of this analysis are presented in Table 5.

**Table 5:** Bonferroni-adjusted pairwise comparisons.

Comparison	Mean differences	Std. error	p	95% CI lower	95% CI upper
SPS1 vs. SPS2	−5.60	1.11	<0.001	−8.33	−2.87
SPS1 vs. SPS3	−17.17	1.04	<0.001	−19.73	−14.61

An examination of Table 5 reveals that all differences between the repeated measures were statistically significant. The Bonferroni-adjusted post-hoc pairwise comparisons indicated a significant difference between SPS1 and SPS2 (*p* < 0.001). Similarly, a significant difference was found between SPS1 and SPS3 (*p* < 0.001). Furthermore, SPS scores at SPS3 were significantly higher than those at SPS2 (*p* < 0.001). This consistent and significant increase in mean scores from SPS1 to SPS2 and finally to SPS3 demonstrates a clear and positive progression in the acquisition of scientific process skills over the course of the intervention.

### 3.2 Impact of graphic organizers on the development of the scientific process skills dimension of DHH students

A series of repeated-measures ANOVAs were conducted to evaluate the impact of graphic organizers on the development of various scientific process skills among students (DHH) over three time points, while controlling for the effect of student attitude. This analysis encompasses five key dimensions: Observation, Classification, Communication, Measurement, Prediction, and Interpretation skills.

#### 3.2.1 Descriptive statistics

The means and standard deviations for all six scientific process skill dimensions across the three time points are presented in Table 6. All dimensions demonstrate a clear and consistent increase from Time 1 (pre-intervention) to Time 3 (post-intervention), indicating a strong positive trend in skill development throughout the graphic organizer intervention period.

**Table 6:** Descriptive statistics for scientific process skills scores across dimensions.

Dimension	Time point	*N*	Mean	Std. deviation	Skewness	Kurtosis
Observation	1	65	9.37	2.45	−0.378	−0.774
	2	65	10.02	2.01	−0.426	−0.799
	3	65	11.85	0.73	−4.890	23.448
Classification	1	65	8.26	2.73	−0.113	−1.288
	2	65	9.18	2.59	−0.393	−1.165
	3	65	11.42	1.22	−1.964	−2.588
Communication	1	65	6.95	2.56	0.440	−0.788
	2	65	8.38	2.77	−0.182	−1.130
	3	65	10.69	1.82	−1.324	1.135
Measurement	1	65	7.18	2.74	0.456	−0.951
	2	65	8.15	2.90	−0.007	−1.238
	3	65	10.42	1.94	−0.876	−0.068
Prediction	1	65	4.45	2.04	1.204	0.183
	2	65	5.42	2.06	0.346	−0.885
	3	65	7.25	1.69	−0.462	−0.391
Interpretation	1	65	2.85	1.34	1.382	0.577
	2	65	3.51	1.37	0.434	−0.823
	3	65	4.62	1.16	−0.196	−0.476

Mauchly’s test of sphericity was not significant for most dimensions (all *p* > 0.05), indicating that the assumption of sphericity was met for these analyses. However, for Observation skills, the data violated the normality assumption, requiring nonparametric analysis using Friedman’s test. The parametric repeated-measures ANOVA results (Table 7) revealed a statistically significant main effect of time on all scientific process skill dimensions. The time factor accounted for varying proportions of variance across dimensions.

**Table 7:** Repeated-measures ANOVA results for scientific process skills (within-subjects effects).

Dimension	Time *F*-value	*p*-value	Partial *η*^2^	Time × attitude *F*-value	*p*-value	Partial *η*^2^
Classification	*F*(2,126) = 6.88	0.001	0.10	*F*(2,126) = 2.79	0.065	0.04
Communication	*F*(2,126) = 4.20	0.017	0.06	*F*(2,126) = 1.16	0.317	0.02
Measurement	*F*(2,126) = 8.01	<0.001	0.11	*F*(2,126) = 5.32	0.006	0.08
Prediction	*F*(2,126) = 10.07	<0.001	0.14	*F*(2,126) = 2.00	0.140	0.03
Interpretation	*F*(2,126) = 6.68	0.002	0.10	*F*(2,126) = 1.09	0.341	0.02

For observation skills, Friedman’s test revealed a statistically significant difference in the distribution of scores across the three time points, *χ*^2^(2) = 61.87, *p* < 0.001. The mean ranks showed a progressive improvement: Observation 1 (Mean Rank = 1.59), Observation 2 (Mean Rank = 1.85), Observation 3 (Mean Rank = 2.56). The pairwise comparisons for observation skills, which used a Bonferroni adjustment, showed a specific pattern. There was no significant (adj. *p* = 0.444) difference between the first and second observations. However, scores from the third observation were significantly higher than those from both the first (adj. *p* < 0.001) and the second observation (adj. *p* < 0.001). This pattern suggests that observation skills exhibited a more pronounced improvement compared to other dimensions over time.

A notable finding emerged regarding the interaction between time and attitude. While most dimensions showed no significant interaction, measurement skills demonstrated a statistically significant time x attitude interaction (*p* = 0.006), indicating that the pattern of improvement in measurement skills differed depending on students’ attitude levels. Classification skills showed a strong trend toward significance (*p* = 0.065), suggesting a potential moderating role of attitude that warrants further investigation. The between-subjects analyses revealed that attitude did not have a significant main effect on the overall level of any scientific process skills (all *p* > 0.05), indicating that students with different attitude levels had similar overall performance across dimensions.

Bonferroni-adjusted pairwise comparisons revealed that all time point differences were statistically significant for most dimensions (*p* < 0.01 for all comparisons). The consistent pattern of significant improvement from Time 1 to Time 2, Time 1 to Time 3, and Time 2 to Time 3 across dimensions demonstrates the progressive and sustained nature of skill development throughout the intervention period (Table 8).

**Table 8:** Summary of Bonferroni-adjusted mean differences across time points.

Dimension	T1-T2 difference	T1-T3 difference	T2-T3 difference
Observation	−0.65	−2.48*	−1.83*
Classification	−0.92*	−3.15*	−2.23*
Communication	−1.43*	−3.74*	−2.31*
Measurement	−0.97*	−3.23*	−2.26*
Prediction	−0.97*	−2.80*	−1.83*
Interpretation	−0.66*	−1.77*	−1.11*

*p < 0.01; For observation skills, T1-T2 difference was not statistically significant in nonparametric analysis.

#### 3.2.2 Differences of scientific process skills in students with hearing loss (DHH) according to their grade level

A mixed-design analysis of variance (ANOVA) was conducted to examine the effects of time (three measurements) and school level on the dependent variable, with attitude included as a covariate. Preliminary assumption testing was conducted to check for normality, sphericity, and homogeneity of variances. Mauchly’s test indicated that the assumption of sphericity had not been violated, *χ*^2^(2) = 0.77, *p* = 0.681. Therefore, sphericity-assumed results are reported. However, Box’s test of equality of covariance matrices was significant (*M* = 24.85, *p* = 0.029), suggesting violation of the homogeneity of covariance assumption. Levene’s test indicated homogeneity of error variances for SPS1 (*p* = 0.199) and SPS2 (*p* = 0.126), but not for SPS3 (*p* = 0.004).

As presented in Table 9, a statistically significant main effect of time was found (partial *η*^2^ = 0.141), demonstrating a significant change in scores over the three measurement points. The effect size suggests a medium effect according to Cohen (1988) criteria. The interaction between time and attitude was not statistically significant (partial *η*^2^ = 0.041) nor was the interaction between time and school level (partial *η*^2^ = 0.019).

**Table 9:** Tests of within-subjects effects.

Source	Sum of Squares	df	MS	*F*
Time	785.88	2	392.94	10.02
Time × attitude	202.50	2	101.25	2.58
Time × school level	90.17	4	22.54	0.58
Error	4,783.31	122	39.21	

There was no statistically significant main effect of school level (partial *η*^2^ = 0.035) or attitude (partial *η*^2^ = 0.038), on the SPS scores (see Table 10).

**Table 10:** Tests of between-subjects effects.

Source	Sum of squares	df	MS	*F*	*p*	Partial *η*^2^	Observed power
Intercept	9,194.79	1	9,194.79	36.02	<0.001	0.371	1.000
Attitude	608.09	1	608.09	2.38	0.128	0.038	0.330
School level	568.41	2	284.20	1.11	0.335	0.035	0.237
Error	15,569.77	61	255.24				

#### 3.2.3 Analysis of qualitative data

**Figure 1:**
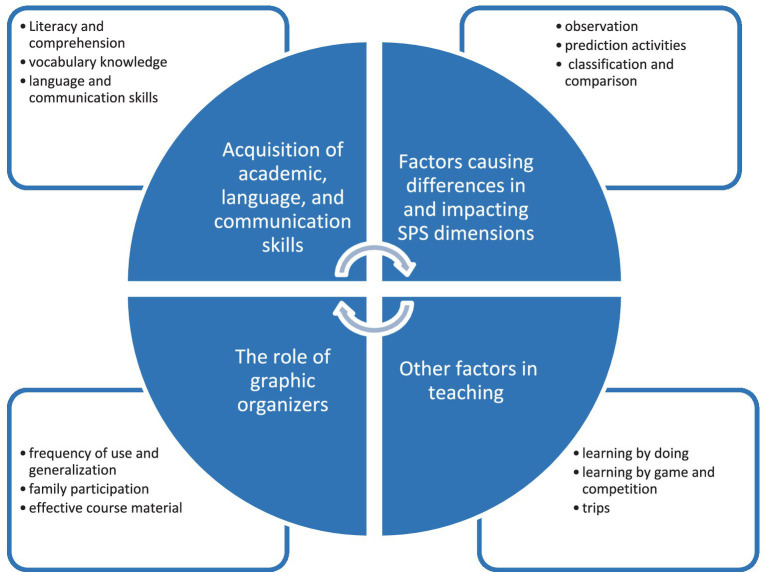
Themes obtained from teachers’ opinions in SPS acquisition.

The interviews that were conducted at times when teachers did not have courses and audio-recorded were converted into written transcripts. The interviews took approximately 15–20 min. Content analysis was carried out (Creswell and Creswell, 2022). As a result of data analysis, four main themes were obtained (Figure 1).

Teachers’ opinions are categorized under four main themes, namely “Acquisition of academic, language, and communication skills,” “Factors causing differences in and impacting SPS dimensions,” “Other factors in teaching,” and “The role of graphic organizers” and their respective sub-themes.

Within the scope of “Acquisition of academic, language, and communication skills,” most teachers stated that vocabulary acquisition is of great importance in the activities (T6, T3, T2, T1). Among teachers who stated that vocabulary knowledge increases students’ participation in the activities and success;

T6: *“… It may* var*y from child to child. Some of my students have a broader vocabulary, they participate in the activities better since they are at different academic levels. Some of my students may have difficulty with this; they cannot even describe things with a single characteristic.”*

Teachers stated that, in terms of communication skills, DHH students prefer sign language in SPS activities while expressing their feelings and opinions and making comments. For example;

T6: *“… some of my students only know sign language, some know a few verbal things. Vocabulary develops depending on the family engagement. Since Özge is in constant communication with her family at home and wears her device carefully, she is more successful in communication. Children are elevated to more different levels when the families are engaged.”*

T1: *“the child hears but cannot comprehend enough, has difficulty in expressing what he understands. It is also highly difficult for them to speak. They prefer sign language.”*

T2: *“The ones who can speak use their sense of hearing but others frequently communicate through touching, sign, and sense of seeing.”*

Within the scope of “The role of graphic organizers,” all teachers stated that they can be used as effective materials, especially in mathematics and science courses. For example;

T2: “*for students with hearing loss, as they are more visual, it is easier to use graphic organizers, especially in the fields of mathematics and science. They understand faster when we use these materials since they can see. It is important to use these tables and graphics correctly, otherwise, misconceptions may occur.*”

T6: *“……we cannot progress without visual materials. These graphic organizers are definitely beneficial. In terms of the permanence of the acquired knowledge.”*

Within the scope of this theme, teachers stated that usage areas of graphic organizers such as home and school have positive effects on generalization. They also mentioned the importance of frequent revisions on learning. For example;

T2: “*I gave a subject to DHH children, fruits, for example. I use these subjects in all courses. Learning a song about fruits in music course. reading exercise. Thus, we make constant revisions. I expect the same support from the family at home. For example, a constant chat about fruits. The child can learn only this way. If the graphic organizers are used at home, it will lead to better learning. Otherwise, the learning is not permanent.”*

T6: “… *different activities can be conducted with graphic organizers. They can definitely use them in daily life when they conduct activities. If the activities are repeated frequently, they start to conduct them with their peers and family members at home.*”

Some teachers reported that while graphic organizers are beneficial, families and students need to be informed about how to implement them (T1, T4, T5). They drew attention to being a role model for the students during the activities, displaying examples and teaching with clues. For example;

T5: “… *I think graphic organizers are beneficial in academic skills. While using graphic organizers, children should be given clues about them and examples should be provided on how to use them. Interpretation, reading are important. If they are taught how to use them, they can utilize them in other courses. We can do it by working on it. It may be very beneficial for the children*.”

T4: “….. *since the hearing impaired’s fundamental education is based on visuals, they generally study on matching, concept matching, and visuals. ... Graphic organizers, of course, support them but not all children can use them as they are hard to apply. ... If examples with graphic organizers are provided, they can use them over time.*”

In “Factors causing differences in and impacting SPS dimensions”, teachers’ opinions about observation, making binary and triple comparisons, grouping skills are provided with examples below:

In classification and comparison in SPS sub-theme, teachers (T1, T3, T2, T6, T4) mentioned that students should be guided and prior knowledge and concept knowledge are important. They expressed that vocabulary knowledge differs among students. For example; T3: “……*They cannot make comparisons, they have difficulty with it. They recognize the differences to a certain extent. It is necessary to give examples for them to realize common and different features. Then, some students can perform it. ....... they cannot make generalizations. .....*”

T1: *“they have difficulty in binary comparisons. First, show their features. Then, we can say the other. They cannot perform both at the same time.”*

T4: “*…The situation can vary according to their academic skills. While some can realize very fast, some cannot realize at all. It is necessary to emphasize that common feature. Students realize it later. This also varies according to the child’s perception. Clues should be provided in different grouping methods. Examples should be displayed. If explained in detail, they can perform it.”*

In observation skills in SPS sub-theme, teachers (T1, T2, T5, T4) drew attention to students’ senses. They stated that while some students participate in visual activities, some students participate in both visual and auditory activities. For example;

*T1: “although all of them are deaf, they are different from each other. Their feelings are different. Their individual characteristics are different, their sense of hearing, time of hearing aid fitting, time of intervention, family support, are different... among children, those who can hear better prefer the sense of hearing and seeing while others prefer the sense of seeing and touching.*”

T2:“*both the activities you carried out and the activities I used in my courses are based on the sense of seeing, body, gestures, and mimics.*”

T5: “*the sense most frequently uısed by children with hearing loss is their eyes. By looking. My student can conduct a binary comparison. He can observe. The most important point is that he needs support while making comparisons. He generally does it with help. As the number of activities increases, the student’s awareness also increases. When we provide the common features as clues at the beginning, they can realize more easily.*”

Regarding how students transfer observation results to each other, T2 drew attention to the importance of concept knowledge. Teachers stated that they use different methods in line with students’ characteristics of language use (T1, T3, T5).

T3: “… *they use sign language. They prefer sign language even though they hear. They don’t want to use written expression.*” T1: “*we can never observe these. They prefer sports, game activities, computers, and smart boards.*”

T5: “*they frequently use sign language in their communication with each other. It is easier for them. They find it harder to talk. Sign language is also practical. They can make their own observation and interpret it. They can generate inferences if they understand the concept. T3: “they dislike mutual dialogues because it is tiring for them.*”

In prediction activities in SPS sub-theme, teachers drew attention to the importance of students’ prior experiences and their interest in the subject matter (T1). Most teachers stated that prediction activities are very difficult for their students. For example;

T2: “… *we encounter difficulties in explaining the predictions. Language skills are important in prediction activities and all of the other activities*...”

T1: “…*if you explain everything in sign language similar to talking to a hearing individual, they can comprehend and make predictions. For example, they can predict the champion in a football match, they can explain their reasoning with 80% accuracy. If they are interested in the subject, of course. Their prior knowledge is of great importance. They can make predictions based on their prior knowledge.*”

T4: “…*it is difficult for them to compare the predictions and real results. They don’t completely understand it.* T5: *“…she couldn’t fully comprehend prediction skills. She said something but she was not completely aware. But if they study harder, they can make predictions.” T3: “….they are aware in terms of prediction skills. they make quick decisions without reasoning.*”

Within the scope of “Other factors in teaching” theme, teachers mentioned the importance of practices related to daily life such as games, trips, observation, and competitions, which enable learning by doing, as well as graphic organizers. For example;

T5: “*trips are very important, for example... we carry out many trips but students can be taken to the market, stationery, and bakery. These are of great importance for them to do things themselves, learn, and socialize*...”

T6: “*Visuals are important for the acquisition of academic skills. It is more important and easier to learn by doing through visual materials. If we can conduct courses in real environments, it will be more beneficial with games and practices. Instruction based on only writing and reading limits children’s education.*”

T2*: “Since the visuals are more developed in academic skills, materials should be designed accordingly. While teaching the structure of the ear, they learn better by touching and seeing when they model the ear with playdough..... Your activities were effective. They included games and competitions. When games and competitions are included in teaching, they learn faster. They can be really curious when technology is actively utilized.*”

T1: “…*learning by doing may be provided through shopping, related to money calculation in mathematics, or for the science course in a natural context. It is important to teach all subjects at experimental level. This is not only related to science, it also applies to other courses.*”

## 4 Discussion

According to the ANOVA results, the use of graphic organizers was associated with statistically significant differences across SPS1, SPS2, and SPS3 scores (partial *η*^2^ = 0.15), indicating a moderate effect size. In addition, the time × attitude interaction was statistically significant at a modest level (partial *η*^2^ = 0.05). Together, these findings suggest a progressive improvement in DHH students’ scientific process skills over time.

This result can be interpreted within the framework of Dual Coding Theory, which posits that learning is enhanced when information is processed through both verbal and visual systems (Paivio, 1986; Clark and Paivio, 1991). In the present study, graphic organizers likely facilitated the integration of visual and linguistic representations, supporting deeper conceptual processing and more effective acquisition of SPS among DHH students. This interpretation strengthens the theoretical explanation of how graphic organizers support SPS development in visually oriented learners.

Although the interaction effect between time and attitude was relatively small, it suggests that students’ affective dispositions may influence the rate of skill development rather than overall achievement levels. This finding highlights the importance of considering motivational and attitudinal factors as complementary components in instructional interventions. Consistent with this result, previous studies have shown that inquiry-based science activities positively influence both achievement and attitudes of DHH students (Kurz et al., 2015; Marchut and Gormally, 2019).

A notable finding was the greater improvement observed in observation skills compared to other SPS dimensions. This may be explained by the visual learning strengths of DHH students, particularly their heightened reliance on visual attention and processing. From a Dual Coding perspective, observation skills are more directly supported by visual representations, which may facilitate encoding and retrieval processes (Marschark and Wauters, 2008). This suggests that visually structured instructional strategies may be especially effective in supporting foundational scientific skills.

In addition to Dual Coding Theory, Cognitive Load Theory provides a complementary explanation for these findings. Learning is optimized when instructional materials reduce extraneous cognitive load and support working memory processing (Sweller, 1988; Sweller et al., 2011). In this study, graphic organizers likely reduced cognitive demands by structuring complex information visually, thereby supporting the organization and integration of scientific concepts. This may have contributed to the observed improvements in SPS performance.

These findings are consistent with previous research. For example, research has shown that visual and auditory–visual instructional materials increase engagement and participation in science learning among DHH students, while sign language–supported science instruction enhances interest and positive attitudes (Mao et al., 2021). Similarly, studies have highlighted challenges in curriculum adaptation, language accessibility, and instructional methods in science education for DHH learners (Mukhopadhyay and Moswela, 2010). These findings align with the present study, which suggests that structured visual tools such as graphic organizers may address some of these instructional barriers.

The finding that school level did not significantly affect SPS development indicates that the intervention was effective across different educational stages. This finding may indicate that when instruction is appropriately adapted to the communication and learning needs of DHH students, developmental differences related to grade level become less influential. Language proficiency, early intervention, and access to appropriate educational support may play a more decisive role than grade level alone (Turnbull et al., 2012; Vázquez, 2019).

Finally, the qualitative findings support and extend the quantitative results. Teachers emphasized the importance of vocabulary development, structured visual supports, and repeated practice in improving SPS. They also highlighted the role of family involvement and the need for consistent use of graphic organizers across contexts. These findings are consistent with previous research emphasizing the importance of multimodal instruction, language support, and experiential learning in DHH education (Freeman et al., 2014; Lomber, 2017). Overall, the results provide empirical support for Dual Coding Theory, suggesting that integrating visual and linguistic elements through graphic organizers enhances both cognitive processing and scientific skill development in DHH learners. However, these interpretations should be considered in light of the study’s limitations, particularly the absence of a control group.

## 5 Conclusion

This study investigated the impact of graphic organizers on the development of scientific process skills (SPS) among students (DHH) in a science course. The findings provide robust and multi-faceted evidence supporting the effectiveness of this instructional tool.

The primary conclusion is that the use of graphic organizers led to a statistically significant and substantial improvement in the overall scientific process skills of DHH students. The repeated-measures ANOVA revealed a strong main effect of time, with a medium effect size (partial *η*^2^ = 0.15). The Bonferroni-adjusted pairwise comparisons confirmed a consistent and progressive increase in the total SPS scores from the pre-test (SPS1) to the mid-test (SPS2) and finally to the post-test (SPS3), with each difference being significant. This clear linear trend demonstrates that the intervention was successful in fostering cumulative growth in scientific reasoning abilities over time.

Delving into the specific skill dimensions, the intervention proved to be effective across all measured components of scientific inquiry. Significant improvements were observed in classification, communication, measurement, prediction, and interpretation skills. A notable finding was the unique developmental pattern of observation skills, where the most significant leap occurred later in the intervention (between SPS2 and SPS3), suggesting that the benefits of graphic organizers for this fundamental skill may require a longer period of exposure to fully manifest. Furthermore, the analysis revealed that student attitude played a moderating role in the development of certain skills. Specifically, a significant time x attitude interaction was found for measurement skills, and a strong trend was observed for classification skills. This indicates that students’ attitudes toward science influenced the *rate* or *pattern* of their skill acquisition in these specific domains, even though attitude did not affect their overall performance levels.

Regarding contextual factors, the study found that the positive impact of graphic organizers was consistent across different grade levels. The mixed-design ANOVA showed no significant interaction between time and school level (elementary, middle, high school), meaning that students at all levels benefited similarly from the intervention. This suggests that graphic organizers are a versatile tool that can be effectively integrated into science curricula for DHH students of various ages and academic stages.

In summary, this study conclusively demonstrates that graphic organizers are a highly effective instructional strategy for promoting the development of scientific process skills in students with hearing loss. The intervention led to significant, progressive gains in both overall scientific proficiency and its constituent skills, proving effective across grade levels. The findings advocate for the intentional and widespread adoption of graphic organizers in science education for DHH students to bridge gaps and foster essential inquiry-based learning skills.

## 6 Limitations

Several limitations of the present study should be acknowledged. First, detailed audiological measures of participants’ functional hearing capacity (e.g., hearing thresholds or speech perception levels) were not included. Instead, hearing profiles were described based on assistive device use and degree of hearing loss (severe and profound). Although these indicators provide indirect insight into auditory characteristics, future research would benefit from incorporating more comprehensive measures.

Second, a single attitude scale was used across a broad age range (9–16 years). Despite procedural adaptations, younger participants may have had difficulty interpreting abstract attitudinal constructs, suggesting the need for developmentally appropriate instruments.

Third, the absence of a control group limits causal interpretations, and the inclusion of participants from different school levels may have introduced heterogeneity. In addition, although the SPS observation form was developed based on the literature and expert review, further evidence of its validity and reliability is needed. The relatively short duration of the intervention also limits the generalizability of the findings. Finally, more detailed reporting of qualitative analysis procedures would further enhance methodological transparency.

## 7 Implications for practice and research

The findings of this study have important implications for educational practice and future research in special and science education.

First, the results emphasize the importance of integrating visual instructional strategies, such as graphic organizers, into science teaching for DHH students. Teachers should be supported through professional development programs focusing on the effective use of structured visual tools.

Second, the findings highlight the need for multimodal instruction that combines visual, linguistic, and experiential elements. Given the diverse communication preferences of DHH students, flexible and inclusive approaches incorporating sign language, written language, and visual supports are essential.

Third, language and vocabulary development play a central role in science learning. Instruction should include explicit teaching of scientific concepts, supported by collaboration among special education teachers, subject teachers, and language specialists.

Fourth, the absence of differences across grade levels suggests that such interventions can be effective across a wide age range. However, individualized adaptations remain necessary due to variability in students’ characteristics.

Fifth, qualitative findings underscore the importance of family involvement in reinforcing learning beyond the classroom.

Finally, future research should employ experimental and longitudinal designs to strengthen causal interpretations and examine long-term effects. Further studies may also explore the role of technology (e.g., digital vs. paper-based tools) and individual differences in shaping learning outcomes.

## Data Availability

The original contributions presented in the study are included in the article/Supplementary material, further inquiries can be directed to the corresponding author.

## References

[ref1] Cannon J. E. Guardino C. Paul P. V. (2022). Deaf and hard of hearing multilingual learners: foundations, strategies, and resources. Routledge. doi: 10.4324/9781003259176 39302100

[ref2] Çiftçi E. (2009). Design, Implementation, and Evaluation of a Computer-Assisted Written Expression skill Development Material for Students With Hearing Impairments (Master’s thesis). Trabzon, Türkiye: Karadeniz Technical University.

[ref3] Clark J. M. Paivio A. (1991). Dual coding theory and education. Educ. Psychol. Rev. 3, 149–210. doi: 10.1007/BF01320076

[ref4] Cohen J. (1988). Statistical Power Analysis for the Behavioral Sciences. 2nd Edn Hillsdale, NJ: Lawrence Erlbaum Associates.

[ref5] Confederación Estatal de Personas Sordas (CNSE). (2021). Manifiesto de la Confederación Estatal de Personas Sordas por la semana internacional de las personas sordas. Available online at: https://cnse.es/index.php/noticias/item/774-semana-internacional-de-las-personas-sordas-2021

[ref6] Creswell J. W. Creswell J. D. (2022). Research Design: Qualitative, Quantitative, and Mixed Methods Approaches. 6th Edn SAGE Publications.

[ref7] Dexter D. D. Hughes C. A. (2011). Graphic organizers and students with learning disabilities: a meta-analysis. Learn. Disabil. Q. 34, 51–72. doi: 10.1177/073194871103400104

[ref8] Ediyanto I. N. Hayashida M. Kawai N. (2018). “A literature study of science process skill toward deaf and hard of hearing students,” in Proceedings of the 1st Annual International Conference on Mathematics, Science and Education (ICoMSE 2017), (Atlantis: Press). doi: 10.2991/icomse-17.2018.23

[ref9] Freeman S. Eddy S. L. McDonough M. Smith M. K. Okoroafor N. Jordt H. . (2014). Active learning increases student performance in science, engineering, and mathematics. Proc. Natl. Acad. Sci. 111, 8410–8415. doi: 10.1073/pnas.1319030111, 24821756 PMC4060654

[ref10] García-Terceño E. M. Greca I. M. Santa Olalla-Mariscal G. Diez-Ojeda M. (2023). The participation of deaf and hard of hearing children in non-formal science activities. Front. Educ. 8:1084373. doi: 10.3389/feduc.2023.1084373

[ref11] Glass G. V. Hopkins K. D. (1996). Statistical Methods in Education and Psychology. 3rd Edn Pearson.

[ref12] Graham S. C. (2012). Deaf education preservice teachers’ perceptions of scientific inquiry and teaching science to deaf and hard of hearing students (doctoral dissertation). University of Tennessee. Available online at: http://trace.tennessee.edu/utk_graddiss/1297

[ref13] Güzel-Özmen R. Bulut A. Peker K. Özbek M. Şentürk I. Taşkın T. (2002). “Science projects in special education classes,” in Paper Presented at the 12th Special Education Congress, (Ankara, Türkiye: Ankara University).

[ref14] Hair J. F. Black W. C. Babin B. J. Anderson R. E. (2019). Multivariate data analysis. 8th Edn Cengage.

[ref15] Hall M. L. Hall W. C. Caselli N. K. (2019). Deaf children need language, not (just) speech. First Lang. 39, 367–395. doi: 10.1177/0142723719834102

[ref16] Howell D. C. (2013). Statistical Methods for Psychology. 8th Edn Belmont, CA: Cengage.

[ref17] Hrastinski I. Wilbur R. B. (2016). Academic achievement of deaf and hard-of-hearing students in an ASL/English bilingual program. J. Deaf. Stud. Deaf. Educ. 21, 156–170. doi: 10.1093/deafed/env072, 26864688 PMC4886322

[ref18] İflazoğlu A. Saban A. (2014). Examination of primary school teacher candidates’ views on the nature of science according to gender and grade level. Gaziantep Univ. J. Soc. Sci. 13, 1121–1135. doi: 10.21547/jss.257188

[ref19] Işıkdoğan Uğurlu N. Kayhan N. Hakkoymaz S. (2026). Mothers’ views on the school-starting process for deaf children. Front. Psychol. 16:1694936. doi: 10.3389/fpsyg.2025.1694936, 41583759 PMC12823789

[ref20] Kaplan K. (2011). Perceptions of basic Astronomy Concepts among Fifth-grade Students with and without Intellectual Disabilities [Master’s thesis]. Bolu, Türkiye: Abant İzzet Baysal University.

[ref21] Knoors H. Marschark M. (2020). Educating deaf learners: updated perspectives. Oxf. Res. Encycl. Educ. doi: 10.1093/acrefore/9780190264093.013.952

[ref22] Kurz K. Schick B. Hauser P. (2015). Deaf children’s science content learning in direct instruction versus interpreted instruction. J. Sci. Educ. Stud. Disabil. 18, 23–37. doi: 10.14448/jsesd.07.0003

[ref23] Lomber S. G. (2017). What is the function of auditory cortex without acoustic input? Cogn. Dev. 42, 49–61. doi: 10.1016/j.cogdev.2017.02.007

[ref24] Mao P. Cai Z. He J. Chen X. Fan X. (2021). The relationship between attitude toward science and academic achievement in science: a three-level meta-analysis. Front. Psychol. 12:784068. doi: 10.3389/fpsyg.2021.784068, 34975676 PMC8716559

[ref25] Marchut A. E. Gormally C. (2019). Inquiry-based laboratories and deaf students. J. Scholarsh. Teach. Learn. 19, 18–31. doi: 10.14434/josotl.v19i4.24469, 28188279

[ref26] Marschark M. Shaver D. M. Nagle K. M. Newman L. A. (2015). Predicting the academic achievement of deaf and hard-of-hearing students from individual, household, communication, and educational factors. Except. Child. 81, 350–369. doi: 10.1177/0014402914563700, 26549890 PMC4634639

[ref27] Marschark M. Wauters L. (2008). “Language comprehension and learning by deaf students,” in Deaf Cognition: Foundations and Outcomes, eds. Marschark M. Hauser P. C. (Oxford: Oxford University Press), 309–350.

[ref28] Mastropieri M. A. Scruggs T. E. Norland J. J. Berkeley S. McDuffie K. Tornquist E. H. . (2006). Differentiated curriculum enhancement. J. Spec. Educ. 40, 130–137. doi: 10.1177/00224669060400030101

[ref29] McGinnis J. R. Kahn S. (2014). “Special needs and talent in science learning,” in Handbook of Research on Science Education, eds. Lederman N. Abell S., vol. 2 (New York, NY: Routledge), 237–259.

[ref30] Ministry of National Education. (2018). Science curriculum. Available online at: https://mufredat.meb.gov.tr/

[ref31] Mukhopadhyay S. Moswela E. (2010). Inside practice of science teachers for students with hearing impairments in Botswana primary schools. Int. J. Spec. Educ. 25, 57–67.

[ref32] Novak J. D. Cañas A. J. (2008). The theory underlying concept maps and how to construct and use them (technical report IHMC CmapTools 2006-01 Rev 01-2008) Florida Institute for Human and Machine Cognition. Available online at: http://cmap.ihmc.us/Publications/ResearchPapers/TheoryUnderlyingConceptMaps.pdf

[ref33] Özcan H. Koca E. (2020). Development of the attitude toward science scale: a validity and reliability study. Eurasian J. Educ. Res. 20, 109–134. doi: 10.14689/ejer.2020.85.6

[ref34] Paivio A. (1986). Mental Representations: A Dual Coding Approach. Oxford: Oxford University Press.

[ref35] Patton M. Q. (2015). Qualitative Research & Evaluation Methods. 4th Edn Thousand Oaks, CA: Sage.

[ref36] Raven S. Whitman G. M. (2019). Science in silence: how educators of the deaf and hard-of-hearing teach science. Res. Sci. Educ. 49, 1001–1012. doi: 10.1007/s11165-019-9847-7

[ref37] Santana R. S. Sofiato C. G. (2018). Estado da arte das pesquisas sobre o ensino de Ciências para estudantes surdos. Práx. Educ. 13, 596–616. doi: 10.5212/PraxEduc.v.13i2.0019

[ref38] Scruggs T. E. Mastropieri M. A. Okolo C. M. (2017). Science and social studies for students with disabilities. Focus. Except. Child. 41, 1–24. doi: 10.17161/foec.v41i2.6835

[ref39] Sweller J. (1988). Cognitive load during problem solving: effects on learning. Cogn. Sci. 12, 257–285. doi: 10.1207/s15516709cog1202_4

[ref40] Sweller J. Ayres P. Kalyuga S. (2011). Cognitive Load Theory. New York, NY: Springer.

[ref41] Turnbull A. P. Turnbull H. R. Wehmeyer M. L. (2012). Exceptional Lives. Boston, MA: Pearson.

[ref42] Vázquez S. (2019). ¿De qué hablamos cuando “hablamos ciencias” en el aula inclusiva con alumnado sordo? Rev. Estud. Leng. Signos 1, 269–288.

