# Supplementary material for: The use of graphic organizers in science education of deaf and hard-of-hearing students
Source: Front Psychol. 2026 May 22;17:1844334. doi: 10.3389/fpsyg.2026.1844334 (PMC13238430; doi:10.3389/fpsyg.2026.1844334)
Supplement: Supplementary file 1 [file Table_1.DOCX]

**Annex-1**

**SCIENTIFIC PROCESS SKILLS CHECKLIST**

|  | **INDICATOR SKILLS** | **Observed** | **Acceptable** | **Need Improvement** |
| --- | --- | --- | --- | --- |
| 1. Observation | 1. Senses used |  |  |  |
|  | 1. Making comparisons |  |  |  |
|  | 1. Realizing differences |  |  |  |
|  | 1. Explaining remarkable features |  |  |  |
| 1. Classification | 1. Finding mutual features |  |  |  |
|  | 1. Describing distinctive features |  |  |  |
|  | 1. Finding different classification methods |  |  |  |
|  | 1. Making binary classification |  |  |  |
| 1. Communication | 1. Knowing subject-appropriate concepts |  |  |  |
|  | 1. Interpreting observations |  |  |  |
|  | 1. Transferring the observation to others in verbal language (narration, verbal presentation, singing a song…) |  |  |  |
|  | 1. Transferring the observation to others without verbal language ( by gestures and mimics, writing, drawing…) |  |  |  |
| 1. Measurement | 1. Dispaying the observed qualities with numbers or symbols |  |  |  |
|  | 1. Making one-to-one matchings |  |  |  |
|  | 1. Recording with data organization formats (charts, tables, graphs, pictures, models...) |  |  |  |
|  | 1. Using different measurement tools or units |  |  |  |
| 1. Prediction | 1. Predicting the difference appropriate to the given condition |  |  |  |
|  | 1. Giving appropriate responses to “What if....” questions |  |  |  |
|  | 1. Explaining differences between predictions and observations |  |  |  |
| 1. Interpretation | 1. Interpreting their observations |  |  |  |
|  | 1. Explaining the reasons for their interpretations |  |  |  |

**Annex-2**

**Teacher Interview Form**

1. To what extent are the activities that were conducted effective in terms of fundamental **observation** skills?
   1. Which senses do your students primarily use?
   2. Can they make comparisons?
   3. Can they describe remarkable features?
2. To what extent are the activities that were conducted effective in terms of fundamental **classification** skills?
   1. Can they describe mutual and different features? (Is it necessary to give examples first? Can they make their own decisions?)
   2. Can they suggest different grouping methods other than the given example?
   3. Can they make classifications based on two features? (For example, considering two features together when asked to line up in accordance with gender and height)
      1. Can they make classifications based on three features?
3. How do they behave when they communicate and explain their observations to others?
   1. Concept knowledge, interpretation of observations, verbal, transfer through sign language, writing...
4. What do you think about utilizing **graphic organizers** (tables, graphs, pictures, charts, concept maps, concept networks, fishbone diagrams) for academic skills acquisition of children with hearing loss?
   1. Displaying qualities with numbers and symbols
   2. Making one-to-one matchings

Do they use them as they are? Can they create them? Can they complete the missing parts?

1. What do you think about your students’ prediction skills?
   1. Predicting the differences appropriate to the given condition
   2. What do you think about their ability to explain the differences and similarities between their predictions and observations?
2. In addition to graphic organizers, which other methods do you recommend to use for acquisition and use of academic skills in daily life by students with hearing loss?

1. Are there any activity suggestions you would like to add to the activities you have observed?
